# Angiogenic Factors in Adult Diffuse Glioma and Their Correlation With Tumor Grade: An Observational Study

**DOI:** 10.7759/cureus.110772

**Published:** 2026-06-13

**Authors:** Vaishali Walke, Soni S, Amit Agrawal, Deepti Joshi, Adesh Shrivastava, Tanya Sharma, Ashwani Tandon

**Affiliations:** 1 Department of Pathology and Laboratory Medicine, All India Institute of Medical Sciences, Bhopal, Bhopal, IND; 2 Department of Pathology, Saraswati Medical College, Unnao, IND; 3 Department of Neurosurgery, All India Institute of Medical Sciences, Bhopal, Bhopal, IND

**Keywords:** adult diffuse glioma, angiogenesis, glioblastoma, microvessel density, vascular endothelial growth factor

## Abstract

Background

Angiogenesis is responsible for the growth, progression, and metastasis of adult diffuse gliomas and other solid tumors. Vascular endothelial growth factor (VEGF) acts as both a pro-angiogenic and a potent angiogenic cytokine, playing a significant role in cell proliferation and endothelial cell permeability. Gliomas are characterized by vascular proliferation, which is responsible for tumor growth and biological behavior and disease outcome. CD34 is a transmembrane phosphoglycoprotein and a pan-endothelial marker that can help examine microvessel density (MVD), a measure used to assess the vascular response to VEGF.

Materials and methods

The present cross-sectional study, conducted on 50 histopathologically proven cases of adult diffuse glioma, was carried out to understand the angiogenic phenomenon by measuring VEGF activity and MVD. These were morphologically graded based on the Central Nervous System (CNS) World Health Organization (WHO) grading system. Immunohistochemical staining for the anti-VEGF antibody was performed to examine the expression of VEGF and scored from 0 to 3. MVD was evaluated through CD34 immunohistochemical staining. Both angiogenic factors were subsequently correlated with the tumor's histological grade.

Results

VEGF expression was observed in 44 of the total 50 cases of adult diffuse glioma. Overexpression was seen to be strongly correlated with higher tumor grade. Most cases revealed higher MVD that ranged from 15 to 105 microvessels/10 high-power fields (hpf). MVD was also compared with tumor grades, exhibiting a higher MVD value with increasing tumor grade in cases of adult diffuse glioma.

Conclusion

Angiogenic response factors such as VEGF expression and MVD played a significant role in adult diffuse glioma progression. Its understanding, therefore, is of vital importance so that the option of personalized therapeutic strategies can be explored and the patient can benefit from the targeted treatment options against VEGF. It is expected to potentially decrease tumor growth and progression and disease burden, thereby improving overall survival outcomes in high-grade glioma and bringing a ray of hope to these patients.

## Introduction

Primary brain tumor is one of the leading causes of cancer-related mortality and has a devastating socioeconomic impact on the patient and their families [1]. Gliomas account for nearly 80% of primary intracranial tumors. Adult diffuse gliomas, the most common type, generally affect cerebral hemispheres and characteristically display a diffuse infiltrative pattern of growth in the surrounding neuropil [2]. Gliomas exhibit a spectrum of vascular proliferations that play a key role in their growth, disease progression, and biological behavior. High-grade gliomas feature higher mitosis, increased microvascular proliferation, and necrosis, with a dismal prognosis, and possess reduced survival outcomes [3]. The angiogenic receptors and factors are upregulated and stimulate tumor oncogenic signaling pathways by activating oncogenes and/or downregulating tumor suppressor genes. Endothelial cell proliferations, most commonly seen as glomeruloid formations, are an important and diagnostic hallmark of high-grade gliomas and the result of numerous key factors such as recruitment, proliferation, and alignment of endothelial cells through their complex interaction and cross-talk with tumor cells. The perivascular growth of diffuse gliomas leads to auto-vascularization as they progress and continue to grow along the pre-existing normal brain microvasculature. Gliomas' increased metabolic demand initially outgrows their blood supply, but in later stages, hypoxia-induced necrosis takes place [4]. Intra-tumoral angiogenesis can be evaluated by assessing microvessel density (MVD), well demonstrated by immunohistochemistry (IHC) with endothelial markers such as CD34. Neovascularization has long been implicated as an important but salient feature of glioma progression and may further lead to unfavorable clinical outcomes in patients with glioma [3]. Vascular endothelial growth factors (VEGF) are strongly linked to neovascularization [4] and a common thread that connects both angiogenesis and tumorigenesis [5]. This potent cytokine is upregulated in the hypoxic zone of gliomas. The binding of VEGF to its receptor initiates the cascade of signaling events which further results in new vessel formation [4]. Despite the recent advancements in treatment modalities like surgical approaches, radiotherapy, and chemotherapeutic agents, high-grade glioma continues to exhibit high mortality and major therapeutic challenges [6]. The present study is conducted to determine the role of angiogenic factors such as VEGF and MVD in adult diffuse glioma and their impact on tumor grade.

This article was previously presented as an oral paper by Dr. Soni S during her time as a postgraduate student at the APCON 2022 Annual Scientific Conference of the Indian Association of Pathologists and Microbiologists (IAPM).

## Materials and methods

This present cross-sectional study was conducted in the Department of Pathology and Laboratory Medicine in collaboration with the Department of Neurosurgery of All India Institute of Medical Sciences, Bhopal, in Bhopal, India, after obtaining approval from the institute's Institutional Human Ethics Committee-Post Graduate Research (IHEC-PGR) (approval number: 2020/PG/July/32).

The study enrolled 50 cases of adult diffuse glioma diagnosed on histopathology between January 2019 and June 2022. Formalin-fixed paraffin-embedded (FFPE) tissue blocks were retrieved and examined for various parameters in the present study. Adult diffuse gliomas graded 2, 3, and 4 based on the Central Nervous System (CNS) World Health Organization (WHO) grading system were included in the present study, whereas pediatric gliomas and patients who had received radiotherapy or chemotherapy before surgical resection were excluded from the study. Patients' demographic findings, such as age, sex, disease duration, anatomical site, and radiological findings, were recorded. Histological typing was based on morphological findings: gliofibrillary background, presence of astrocytic cells displaying round to oval pleomorphic nuclei, and indistinct cell borders traversing thin-walled capillary blood vessels were the classifiers of tumors of astrocytic nature, while the features consisting of round to polygonal cells with clear moderate cytoplasm, centric round pleomorphic nuclei having fine chromatin giving a fried egg appearance, and chicken-wire blood vessels were the classifiers of those oligodendroglial in nature. Grading was performed as per the CNS WHO grading system based on only the morphological criteria such as the presence of hypercellularity, nuclear pleomorphism, mitosis, microvascular proliferation, and necrosis [3,7].

A complete molecular workup was not available in all cases and hence was not a parameter of glioma characterization. One representative FFPE tissue block was selected for IHC for the following markers: VEGF (clone RBT-VEGF), a rabbit polyclonal antibody, and CD34 (clone CD34 QBEnd/10), a mouse monoclonal antibody. The tissue processing protocols are the standard ones. Antigen retrieval was performed using MERS (Multi Epitope Retrieval System) (PathnSitu Biotechnologies, Secunderabad, India) for a period of 30-45 minutes in a Tris-ethylenediaminetetraacetic acid (EDTA) buffer at pH 9.0. IHC staining was performed using the manual staining kit PolyExcel HRP/DAB Detection System (PathnSitu Biotechnologies, Secunderabad, India), with the kit contents comprising the PolyExcel peroxidase quencher (H2O2), PolyExcel target binder, PolyExcel poly-horseradish peroxidase (poly-HRP), PolyExcel Stunn DAB substrate buffer, and PolyExcel Stunn DAB chromogen, while ready-to-use primary antibodies such as VEGF and CD34 were utilized. The IHC expression of both VEGF and CD34 was evaluated by two independent observers, and a consensus score was obtained once they agreed upon it. The IHC expression was determined based on the preset cutoff values. The percentage of stained cells was noted as a continuous variable. For VEGF, the cytoplasmic expression was scored as 0 to 3+, according to the criteria laid down by Raica et al. [8]. The placental tissue served as a positive control. The expression of VEGF was examined across all types and grades of adult diffuse glioma. MVD was evaluated by staining CD34, a pan-endothelial marker, which was screened at 10× magnification, and 10 regions with the highest number of evident microvessels were read using the Leica DM750 microscope (Leica Microsystems, Wetzlar, Germany). The microvessel in the non-neoplastic brain adjacent to the tumor was taken as the internal positive control, while the tissue in which the primary antibody was omitted was considered the negative control.

Statistical analysis

Statistical analysis was performed using IBM SPSS Statistics for Windows, Version 25.0 (IBM Corp., Armonk, New York, United States). Data were entered, cleaned, and analyzed systematically. Descriptive statistics such as mean, standard deviation, frequency, and percentage were used to summarize the demographic and clinical characteristics of the study participants. Continuous variables were expressed as mean±standard deviation (SD). For inferential statistics, categorical variables were compared using Fisher's exact test, as several cells in the contingency tables had expected frequencies of less than five. Correlation between angiogenic markers and WHO grades was assessed for statistical significance. A p-value of less than 0.05 was considered statistically significant.

## Results

In the present cross-sectional analysis, the mean age of patients at the time of diagnosis was 43.43 years, ranging from 21 to 65 years, with a male-to-female ratio of 1.5:1. The frontal lobe was the most commonly involved anatomical site, followed by the temporal lobe. Midline location was observed in only three out of the total 50 patients with adult diffuse glioma (Table 1).

**Table 1:** Demographic details of the patients with adult diffuse glioma WHO: World Health Organization

	Frequency (n)	Percentage (%)
Age (years)		
21-40	18	36%
41-60	25	50%
>60	7	14%
Mean age	43.43±11.1	-
Gender		
Male	30	60%
Female	20	40%
Tumor site		
Frontal lobe	21	42%
Temporal lobe	11	22%
Others/midline	18	36%
WHO grade		
Grade 2	12	24%
Grade 3	7	14%
Grade 4	31	62%

All cases were classified and assigned CNS WHO grades based on morphological features. Most cases (n=31) belonged to CNS WHO grade 4, followed by grade 2 (n=12) and grade 3 (n=7). Among the 12 grade 2 adult diffuse gliomas, five morphologically resembled diffuse astrocytoma, while seven were oligodendrogliomas. Of the seven grade 3 gliomas, five were anaplastic astrocytoma and two anaplastic oligodendroglioma. The rest, 31, were classified as grade 4 glioma.

**Figure 1:**
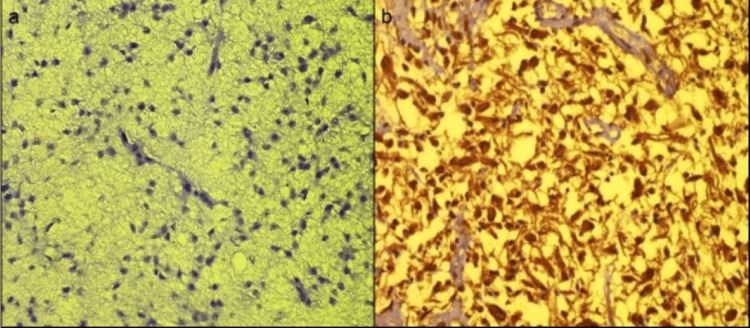
VEGF expression in adult diffuse glioma (a) Diffuse astrocytoma revealing a negative VEGF expression (score 0) (VEGF IHC: 20×). (b) High-grade glioma displaying a strong VEGF expression (score 3) (VEGF IHC: 20×) VEGF: vascular endothelial growth factor; IHC: immunohistochemistry

VEGF expression was evaluated and scored in all 50 cases of adult diffuse glioma. No VEGF expression was observed in 12% of cases, while scores of 1+, 2+, and 3+ were observed in 18%, 28%, and 42% of cases, respectively. For further analysis, VEGF expression was categorized as low expression (scores 0 and 1+) and high expression (scores 2+ and 3+). Most cases (n=35; 70%) demonstrated high VEGF expression. Low VEGF scores were predominantly observed in CNS WHO grade 2 tumors, including both astrocytomas and oligodendrogliomas (Figure 1a). The highest VEGF score (3+) was most frequently observed in grade 4 adult diffuse gliomas, accounting for 16 cases (51.61%), followed by anaplastic astrocytoma and oligodendrogliomas (Figure 1b; Table 2).

**Table 2:** VEGF expression in adult diffuse glioma and its various morphological types * indicates that the value is less than 0.05 (significant) VEGF: vascular endothelial growth factor; CNS: Central Nervous System; WHO: World Health Organization

VEGF score	CNS WHO grade 2 astrocytoma (n=5)	CNS WHO grade 2 oligodendroglioma (n=7)	CNS WHO grade 3 astrocytoma (n=5)	CNS WHO grade 3 oligodendroglioma (n=2)	CNS WHO grade 4 (n=31)	Total (n=50)	P-value
0	0 (0%)	4 (57.14%)	0 (0%)	0 (0%)	2 (6.45%)	6 (12%)	0.042^*^ Fisher's exact test
1+	1 (20%)	2 (28.57%)	2 (40%)	0 (0%)	4 (12.90%)	9 (18%)	
2+	2 (40%)	1 (14.29%)	1 (20%)	1 (50%)	9 (29.03%)	14 (28%)	
3+	2 (40%)	0 (0%)	2 (40%)	1 (50%)	16 (51.61%)	21 (42%)	

Comparison of VEGF expression with CNS WHO grades demonstrated a statistically significant difference between grade 2 and grade 4 tumors (Table 3). Furthermore, comparison of low VEGF expression (scores 0 and 1+) versus high VEGF expression (scores 2+ and 3+) between low-grade gliomas (CNS WHO grade 2) and high-grade gliomas (CNS WHO grades 3 and 4) also showed statistical significance (p=0.044).

**Table 3:** VEGF expression score in adult diffuse glioma: comparison with tumor grade * indicates that the value is less than 0.05 (significant) VEGF: vascular endothelial growth factor; CNS: Central Nervous System; WHO: World Health Organization

VEGF expression score	CNS WHO grade 2 glioma (n=12)	CNS WHO grade 3 glioma (n=7)	CNS WHO grade 4 glioma (n=31)	Total (n=50)	P-value
0	4 (33.33%)	0 (0%)	2 (6.45%)	6 (12%)	Grade 2 vs. 4: 0.05^* ^Fisher's exact test
1+	3 (25%)	2 (28.57%)	4 (12.90%)	9 (18%)	
2+	3 (25%)	2 (28.57%)	9 (29.03%)	14 (28%)	
3+	2 (16.67%)	3 (42.86%)	16 (51.61%)	21 (42%)	

**Figure 2:**
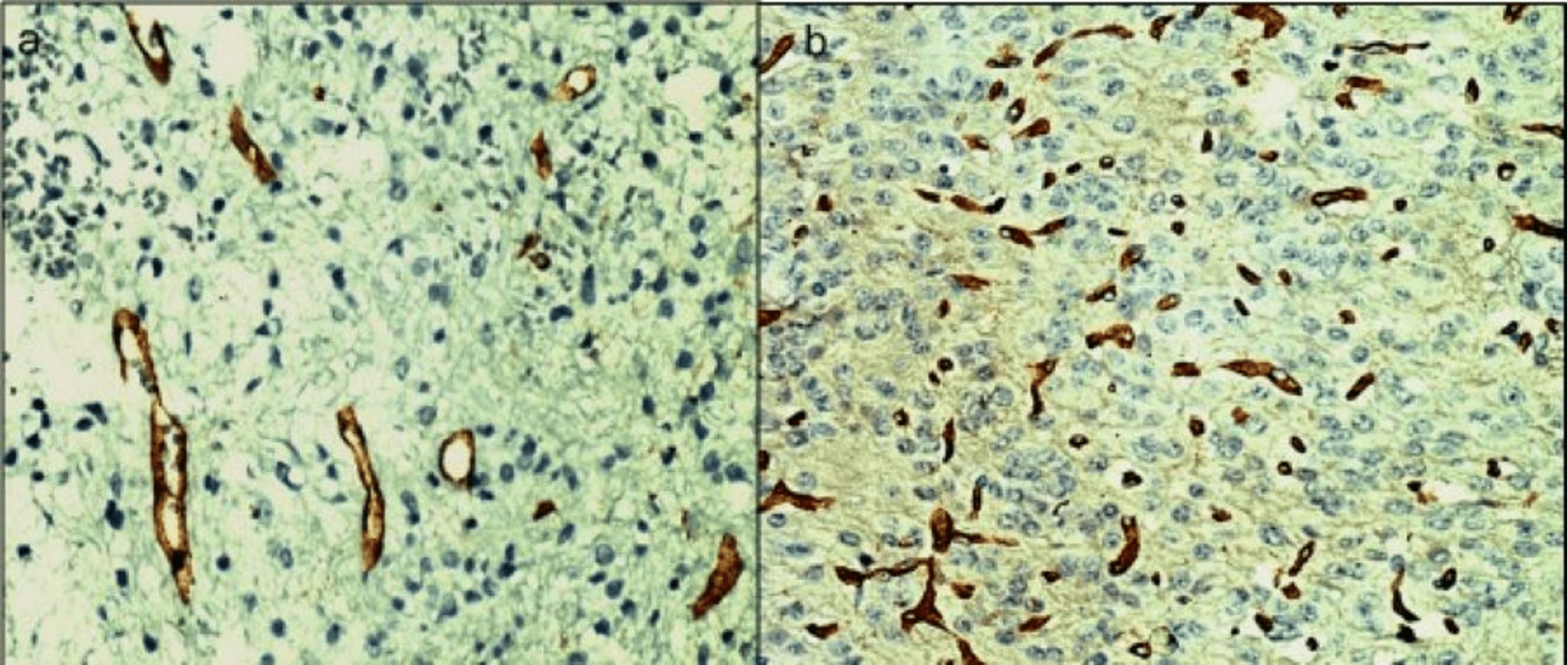
Microvessel density in adult diffuse glioma (a) Grade 2 diffuse astrocytoma showing a low microvessel density count, less than 10 microvessels/hpf (CD34 IHC: 10×). (b) High-grade glioma exhibiting an increased microvessel density, more than 20 microvessels/hpf (CD34 IHC: 10×) IHC: immunohistochemistry; hpf: high-power field

MVD, another angiogenic marker, was also evaluated in the present study. The observed MVD ranged from 15 to 105 microvessels/10 high-power fields (hpf), with a mean value of 60 microvessels/10 hpf. MVD was further categorized into low and high groups using a cutoff value of 50 microvessels/10 hpf. Most patients (n=30; 60%) demonstrated MVD greater than 50 microvessels/10 hpf. Comparison of low and high MVD with tumor grades revealed a statistically significant association. Additionally, MVD showed a positive correlation with increasing tumor grade, with higher MVD values observed in higher-grade tumors (Figure 2a-2b; Table 4).

**Table 4:** Microvessel density and its correlation with tumor grade in adult diffuse glioma * indicates that the value is less than 0.05 (significant) hpf: high-power field

Microvessel density	Grade 2 (n=12)	Grade 3 (n=7)	Grade 4 (n=31)	Total (n=50)	P-value
Low (<50 microvessels/10 hpf)	11 (91.67%)	3 (42.86%)	6 (19.35%)	20 (40%)	<0.0001^*^ Fisher's exact test
High (>50 microvessels/10 hpf)	1 (8.33%)	4 (57.14%)	25 (80.65%)	30 (60%)	

## Discussion

Primary brain tumors are the most prevalent causes of cancer-related death and constitute 40-50% of all intracranial tumors, with the majority being gliomas [9]. Adult diffuse gliomas are defined as infiltrating glial tumors of the central nervous system that infiltrate the surrounding neuropil and stand as the most aggressive tumors [10]. The present study of adult diffuse glioma displayed a wide age range, varying from 21 to 65 years. The maximum number of patients, 31.5%, were in the 41-50-year age group. This age spans all the grades of tumors included in the category of adult diffuse glioma. The average age of patients with low-grade glioma (CNS WHO grades 1 and 2) was 34 years, while it was 56 years for high-grade glioma patients (CNS WHO grades 3 and 4). Similar findings were observed by Liao et al. [11], who mentioned a mean age of 41 years in their study. Male patients (57%) were considered to be affected 1.34 times more than their female counterparts (43%), and the biological behavior is said to be worse in males [12]. In the present study, low-grade gliomas consist of CNS WHO grade 1-2 tumors, while high-grade gliomas comprise CNS WHO grade 3-4 tumors. Low-grade gliomas exhibit better prognosis and are relatively slow-growing [13]. The supratentorial compartment and frontal lobe were the most common locations, but it can develop along the midline structures like the brainstem and spinal cord. In the present study, the tumor was located in the frontal lobe in 21 patients, 11 had involvement of the temporal lobe, and three were in the frontotemporal area, while the midline and posterior-frontal lobe were the sites for the tumor in three patients each. The frontal lobe being the commonest site, as noted in the current study, was also emphasized by Shrestha et al. [14], Thambi et al. [15], and Larjavaara et al. [16]. Angiogenesis, the hallmark of cancer, plays a crucial role in the complex mechanism of tumor development. The degree of angiogenesis affects the tumor's biology, invasiveness, and aggressive behavior. VEGF, an endothelial cell-specific growth factor, stimulates angiogenesis as well as vascular permeability and is responsible for the process of neovascularization that plays a pivotal role in angiogenesis and tumorigenesis in high-grade gliomas. It also promotes the proliferation, survival, and migration of endothelial cells. Binding of VEGF to its receptors starts a signaling cascade event in hypoxic areas that ultimately leads to neovascularization, vascular hyperplasia, and the creation of glomeruloid structures [17]. To understand the role of VEGF in the angiogenesis of adult diffuse glioma in the present population, the expression of VEGF was studied. The average VEGF expression was 29.83±32.24% for low-grade gliomas and 53.05±32.95% for high-grade gliomas. The literature mentions the quantitative and qualitative investigations of VEGF levels in patients with gliomas. The elevated VEGF levels in various studies were correlated with the tumor's aggressive behavior and poor prognosis [9]. Seyedmirzaei et al., in a multivariate analysis, compared the serum levels of VEGF in low- and high-grade gliomas performed using three different modalities, that is, IHC, immunofluorescence, and real-time quantitative reverse transcription polymerase chain reaction (qRT-PCR) [9]. Loureiro et al. [17] evaluated VEGF using IHC in both grade 3 and 4 gliomas, while Zhou et al. [18] assessed it by real-time qRT-PCR on tissue samples. Similarly, Lafuente et al. [19] performed IHC in low- and high-grade gliomas. Awasthi et al. [20] also studied VEGF using IHC in low- and high-grade gliomas and concluded that there was a significant difference between these two groups (p=0.02). Pang et al., in another study, performed IHC for VEGF-A in WHO grade 2, 3, and 4 gliomas and concluded that 86% of gliomas displayed VEGF reactivity. The comparison of VEGF expression by IHC indicated significantly high VEGF in high-grade glioma, while the serum levels of VEGF in both low- and high-grade gliomas were statistically insignificant [21]. In another study, Vokuda et al. evaluated the VEGF levels in glioma patients and healthy controls. They found a highly significant association (p<0.00001) which demonstrated that high VEGF was associated with microvascular proliferations and necrosis in glioma [22]. The observations in the present study, regarding VEGF expression in adult diffuse gliomas, were in concordance with the abovementioned studies. Heinke et al. demonstrated that VEGF levels were associated with subsequent risk of disease progression in glioma (p<0.05) [23]. The VEGF expression was categorized from 0 to 3+ and further classified as low scores (0 to 1+) and high scores (2+ to 3+). The low VEGF expression was evident in grade 2 oligodendroglioma as compared to grade 3. Its expression was equivocal in grade 2 and 3 diffuse astrocytoma. The high expression score of VEGF was noted in most cases of grade 4 diffuse gliomas (25/31), while few (6/31) showed lower expression. The authors in the present study have furthermore classified VEGF expression as low and high depending on its expression score and compared it with CNS WHO grades as low and high grade; the comparison was found to be statistically significant, which inferred that in adult diffuse glioma with rising tumor grade, the expression of VEGF also increases (higher scores) (Table 5). This further substantiates that VEGF is an angiogenic factor that plays a significant role in disease progression and can be responsible for its poor outcome. Our study concurred with the observations made by Vokuda et al. [22]. The evaluation of this important angiogenic factor in adult diffuse glioma can provide potentially useful information in line with personalized medicine in which patients can benefit from anti-VEGF targeted therapy that can also help in improving their overall survival. 

**Table 5:** VEGF expression in adult diffuse glioma: a study comparison VEGF: vascular endothelial growth factor; CNS: Central Nervous System; WHO: World Health Organization

CNS WHO grade	Vokuda et al. (2017) [22] (N=60)	Heinke et al. (2013) [23] (N=99)	Present study (2022) (N=50)
Grade 2	N=15	N=25	N=12
	Mean VEGF: 100%	Mean VEGF: 43.1%	Mean VEGF: 30%
	7 cases: 0, 1+ (low score)	Scoring not done	7 cases: 0 to 1+ (low score)
	8 cases: 2+, 3+ (high score)		5 cases: 2+ to 3+ (high score)
Grade 3	N=15	N=16	N=7
	Mean VEGF: 100%	Mean VEGF: 64%	Mean VEGF: 27%
	1 case: 0, 1+ (low score)	Score: not done	2 cases: 0 to 1+ (low score)
	14 cases: 2+, 3+ (high score)		5 cases: 2+ to 3+ (high score)
Grade 4	N=30	N=12	N=31
	Mean VEGF: 93.33%	Mean VEGF: 75%	Mean VEGF: 57%
	4 cases: 0, 1+ (low score)	Score: not done	6 cases: 0 to 1+ (low score)
	26 cases: 2+, 3+ (high score)		25 cases: 2+ to 3+ (high score)
Low vs. high score	-	0.012 (<0.05)	0.042 (<0.05) Fisher's exact test

Tumor angiogenesis, as one of the vital factors in tumor biology, can also be studied by understanding the presence of intra-tumoral MVD. MVD can be evaluated by both quantitative and qualitative methods and is considered an important and reliable predictor of tumor growth and metastasis and patient survival. The bizarre neovascularization is the hallmark of high-grade gliomas (glioblastoma) and reflects the phenomenon of neoangiogenesis [24,25]. Jha et al. applied IHC markers, such as CD34, CD31, and vWF, in 24 patients with glioblastoma to evaluate MVD and concluded that CD34 is one of the best endothelial markers [24]. The MVD in the current study was examined on the 50 cases of adult diffuse glioma by IHC using CD34 as an endothelial marker and consequently compared across all grades of adult diffuse glioma. The MVD varies in the range of 15-105 microvessels/10 hpf with a mean count of 60 microvessels/10 hpf. The range of MVD in low-grade tumors (CNS WHO grade 2) varied from 15 to 50.5 microvessels/10 hpf, while in high-grade gliomas (CNS WHO grades 3 and 4), it ranged from 25 to 150 microvessels/10 hpf. The authors divided the 50 cases of gliomas into two groups based on the MVD cutoff of 50 microvessels/10 hpf. The study also compared the MVD with tumor grade. MVD of less than 50 microvessels/10 hpf was considered a low score, whereas >50 microvessels/10 hpf MVD was taken as a high MVD score. When this was compared across all histological grades in glioma, it was observed to be highly significant (CNS WHO grades 2 and 3 vs. grade 4). The study revealed an elevated score of MVD proportional to the increasing tumor grade in patients with adult diffuse glioma. The authors experimented with applying different endothelial IHC markers and different cutoffs to study MVD in glioma. Abdulrauf et al. [26], Birlik et al. [27], and Saffar et al. [28] studied CD31, CD105, and factor VIII, while Fan et al. [29] studied the combination of these markers. They examined the association of MVD with different tumor grades in glioma which was statistically significant (p<0.0001) (Table 6). This neoangiogenic event is considered to be of great clinical significance and is linked to disease progression and poor outcomes. The degree of angiogenesis reflects upon tumor biology and its invasive potential and aggressive behavior. The development of anti-angiogenic therapeutic modalities for the treatment of gliomas requires more studies of the angiogenic factors [23,29].

**Table 6:** MVD in adult diffuse glioma: a study comparison IHC: immunohistochemistry; MVD: microvessel density; hpf: high-power field; LG: low-grade; LGG: low-grade glioma; HGG: high-grade glioma

Study (year)	Endothelial marker (IHC)	Sample size	Association with tumor grade	Cutoff for MVD	Association with survival
Abdulrauf et al. (1998) [26]	Factor VIII	N=74	Higher MVD associated with increasing tumor grade	7 microvessels/20 hpf	MVD: independent prognostic indicator of survival in fibrillary LG astrocytoma
Birlik et al. (2006) [27]	CD31	N=70	LGG vs. HGG (p=0.0001)	70 microvessels/40 hpf	MVD: prognostic indicator in adult astrocytoma
Saffar et al. (2018) [28]	CD105	N=48	Higher-grade glioma revealed a higher value of MVD	-	-
Fan et al. (2019) [29]	CD31, CD34, CD105, and factor VIII	N=536	Higher-grade gliomas associated with high MVD count (p=0.0001)	52 microvessels/10 hpf	Higher MVD associated with worse overall survival
Present study (2022)	CD34	N=50	Higher MVD count associated with increasing tumor grade (p=0.0001)	50 microvessels/10 hpf	Survival not estimated

Limitations

This study is limited by its relatively small sample size (n=50), which may restrict the generalizability of the findings. Additionally, being a tertiary center, a higher proportion of high-grade gliomas were observed compared to low-grade cases. Furthermore, complete molecular characterization (e.g., isocitrate dehydrogenase (IDH) mutation, 1p/19q codeletion), which is now an integral part of the CNS WHO 2021 classification, was not performed for all cases due to resource constraints. This may affect the definitive subtyping in some instances. The cross-sectional design prevents association with the survival outcome. The study was conducted at a single center, which may limit generalizability. Furthermore, the absence of multivariate analysis makes it difficult to determine whether VEGF and MVD are independent predictors of tumor grade. 

## Conclusions

A comprehensive understanding of the vascular microenvironment and the mechanisms underlying disease progression in adult diffuse glioma is essential for the development of novel and effective targeted therapies. In the present study, the degree of angiogenesis, assessed by VEGF expression and MVD, demonstrated their potential utility as important angiogenic biomarkers. These markers may play a crucial role in the development of more effective and resistance-free targeted therapeutic strategies. Patients, particularly those with high-grade gliomas, may benefit from personalized treatment approaches based on angiogenic profiling, thereby potentially improving overall survival and clinical outcomes.
